# Wood-Derived Dietary Fibers Promote Beneficial Human Gut Microbiota

**DOI:** 10.1128/mSphere.00554-18

**Published:** 2019-01-23

**Authors:** Sabina Leanti La Rosa, Vasiliki Kachrimanidou, Fanny Buffetto, Phillip B. Pope, Nicholas A. Pudlo, Eric C. Martens, Robert A. Rastall, Glenn R. Gibson, Bjørge Westereng

**Affiliations:** a Faculty of Chemistry, Biotechnology and Food Science, Norwegian University of Life Sciences, Aas, Norway; b Department of Food and Nutritional Sciences, University of Reading, Reading, United Kingdom; c Department of Microbiology and Immunology, University of Michigan, Ann Arbor, Michigan, USA; University of California, Davis

**Keywords:** carbohydrate-active enzymes, dietary fibers, gut microbiota, hemicellulose, *in vitro* fecal fermentation, prebiotics, short-chain fatty acids

## Abstract

Woody biomass is a sustainable and virtually unlimited source of hemicellulosic polysaccharides. The predominant hemicelluloses in softwood and hardwood are galactoglucomannan (GGM) and arabinoglucuronoxylan (AGX), respectively. Based on the structure similarity with common dietary fibers, GGM and AGX may be postulated to have prebiotic properties, conferring a health benefit on the host through specific modulation of the gut microbiota. In this study, we evaluated the prebiotic potential of acetylated GGM (AcGGM) and highly acetylated AGX (AcAGX) obtained from Norwegian lignocellulosic feedstocks *in vitro*. In pure culture, both substrates selectively promoted the growth of *Bifidobacterium*, *Lactobacillus*, and *Bacteroides* species in a manner consistent with the presence of genetic loci for the utilization of β-manno-oligosaccharides/β-mannans and xylo-oligosaccharides/xylans. The prebiotic potential of AcGGM and AcAGX was further assessed in a pH-controlled batch culture fermentation system inoculated with healthy adult human feces. Results were compared with those obtained with a commercial fructo-oligosaccharide (FOS) mixture. Similarly to FOS, both substrates significantly increased (*P < *0.05) the *Bifidobacterium* population. Other bacterial groups enumerated were unaffected with the exception of an increase in the growth of members of the *Bacteroides*-*Prevotella* group, Faecalibacterium prausnitzii, and clostridial cluster IX (*P < *0.05). Compared to the other substrates, AcGGM promoted butyrogenic fermentation whereas AcAGX was more propiogenic. Although further *in vivo* confirmation is necessary, these results demonstrate that both AcGGM and AcAGX from lignocellulosic feedstocks can be used to direct the promotion of beneficial bacteria, thus exhibiting a promising prebiotic ability to improve or restore gut health.

**IMPORTANCE** The architecture of the gut bacterial ecosystem has a profound effect on the physiology and well-being of the host. Modulation of the gut microbiota and the intestinal microenvironment via administration of prebiotics represents a valuable strategy to promote host health. This work provides insights into the ability of two novel wood-derived preparations, AcGGM and AcAGX, to influence human gut microbiota composition and activity. These compounds were selectively fermented by commensal bacteria such as *Bifidobacterium*, *Bacteroides*-*Prevotella*, F. prausnitzii, and clostridial cluster IX spp. This promoted the microbial synthesis of acetate, propionate, and butyrate, which are beneﬁcial to the microbial ecosystem and host colonic epithelial cells. Thus, our results demonstrate potential prebiotic properties for both AcGGM and AcAGX from lignocellulosic feedstocks. These findings represent pivotal requirements for rationally designing intervention strategies based on the dietary supplementation of AcGGM and AcAGX to improve or restore gut health.

## Abstract (precis)

The architecture of the gut bacterial ecosystem has a profound effect on the physiology and well-being of the host. Modulation of the gut microbiota and the intestinal microenvironment via administration of prebiotics represents a valuable strategy to promote host health. This work provides insights into the ability of two novel wood-derived preparations, AcGGM and AcAGX, to influence human gut microbiota composition and activity. These compounds were selectively fermented by commensal bacteria such as *Bifidobacterium*, *Bacteroides*-*Prevotella*, F. prausnitzii, and clostridial cluster IX spp. This promoted the microbial synthesis of acetate, propionate, and butyrate, which are beneﬁcial to the microbial ecosystem and host colonic epithelial cells. Thus, our results demonstrate potential prebiotic properties for both AcGGM and AcAGX from lignocellulosic feedstocks. These findings represent pivotal requirements for rationally designing intervention strategies based on the dietary supplementation of AcGGM and AcAGX to improve or restore gut health.

## INTRODUCTION

The community of microbes inhabiting the human gastrointestinal tract consists of a large variety of bacterial species that collectively influence numerous aspects of human nutrition and health (1). *Firmicutes*, *Bacteroidetes*, and *Actinobacteria* phyla are typically dominant, with specific symbiotic members supplying an arsenal of carbohydrate-active enzymes (CAZymes) for the saccharification and fermentation of otherwise indigestible dietary polysaccharides to short-chain fatty acids (SCFAs) (2). Accumulation of SCFAs in the gut is thought to benefit the host by promoting immune system development and improving local and systemic health. For example, butyrate is the primary energy source for colonocytes and is essential for the regulation of cell growth and differentiation (3); propionate is metabolized in the liver, where it promotes gluconeogenesis and regulates cholesterol synthesis (4); and acetate enters the systemic circulation and is used to generate ATP in muscle tissue and for lipogenesis (5).

Prebiotics are non-host-digestible food ingredients which can shift the gut microbiota composition and metabolic activities, eventually conferring health benefits to the host (6). Currently established prebiotics are inulin-derived fructans (inulin and fructooligosaccharides [FOS]), galactans (galactooligosaccharides [GOS]), and lactulose (6). These aforementioned compounds induce a selective enhancement of *Bifidobacterium* and *Lactobacillus* populations, which are considered beneficial bacteria and therefore represent a common benchmark for prebiotic action (6). Recently, it has been recognized that the prebiotic effect can go beyond stimulation of these two microbial targets and that further health benefits may be derived from a broader range of beneficial bacteria, including SCFA-producing taxa (6). In addition, considering the limited number of available prebiotics, there is a large potential for the generation of novel candidates (7). These new prebiotics can be manufactured from readily available, renewable carbohydrate sources, i.e., plant cell walls.

**FIG 1:**
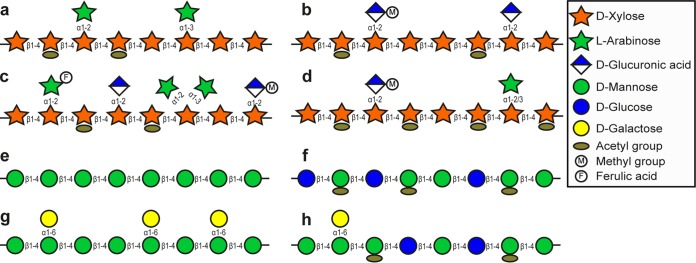
Schematic representation of xylans and β-mannans present in cereals, vegetables, legume seeds, and wood biomass. The shown substrates are as follows: a, wheat arabinoxylan; b, hardwood glucuronoxylan; c, glucuronoarabinoxylan from cereals; d, highly acetylated arabinoglucuronoxylan from Norwegian birch wood; e, unsubstituted mannan from aloe vera; f, konjac glucomannan; g, carob galactomannan; h, acetylated galactoglucomannan from Norwegian spruce wood.

Hemicelluloses represent the second most abundant class of plant polysaccharides, with xylans and mannans being the most prevalent (8, 9). Xylans are major components of commonly consumed cereal grains, fruits, and vegetables, where they may constitute more than 30% of the dry weight. Xylans consist of a β-1,4-linked xylose main chain that can be substituted with arabinose linked at xylose *O*-2 and/or *O*-3 (arabinoxylan), unmethylated and/or methylated glucuronic acid linked to the *O*-2 position (glucuronoxylan, glucuronarabinooxylan), acetic acid in the *O*-2 and/or *O*-3 position, or ferulic acid esterified on the *O*-5 of the substituted arabinose (8) (Fig. 1a to c). β-Mannans are polysaccharides found in certain nuts, beans, and legume seeds, and some are widely utilized as food-thickening agents. The homopolymeric mannan backbone consists of a linear chain of β-1,4-linked mannose residues (Fig. 1e) and can be branched with α-1,6-linked galactosyl groups in galactomannans (Fig. 1g) (8). Glucomannan contains a combination of β-1,4-linked mannose and glucose sugars as a backbone and can be acetylated at positions *O*-2, *O*-3, and/or *O*-6 (Fig. 1f) (8). Strikingly, β-mannans and xylans from woody biomass share high structural similarities with those from food sources. Thus, galactoglucomannan from Norwegian spruce wood has a main chain, which is made up of randomly distributed mannose and glucose sugars, substituted with acetyl groups and galactose units linked to the mannose units (Fig. 1h) (10). Spruce galactoglucomannan alone shows the same structural variations as the different types of mannans listed above (Fig. 1h). Arabinoglucuronoxylan from Norwegian birch wood includes a main chain ornamented with acetyl groups, arabinose, and methylated glucuronic acid (Fig. 1d) (11). Notably, the presence of acetylations and various substituents requires an extensive set of CAZymes for the complete removal of side chains, thus rendering the wood-based oligosaccharides highly selective for microbes equipped with a suitable complex utilization apparatus. Moreover, they may provide increased bioavailability of acetic acid in the colon, where extracellular microbial esterases can release the acetyl groups (12).

The extraction of hemicelluloses from different forms of woody biomass offers the possibility of valorizing these abundant and virtually unlimited compounds and provides novel xylo-oligosaccharides (XOS)/xylans or β-manno-oligosaccharides (β-MOS)/β-mannans with various degrees of polymerization (DP) and substitutions (10, 11). Even though there has been increasing interest in the structure and utilization of XOS/xylans and β-MOS/β-mannans manufactured from woody biomass, limited information is available on their use as a potential resource with health-promoting properties. Previous studies investigating the prebiotic potential of XOS employed fractions obtained from cereals and fruit waste streams (corn cobs [13], corn straw [14], olive [15], and rice husk [16]), brewers’ spent grains, and eucalyptus wood (17). The XOS mentioned above selectively stimulated bifidobacteria, which led to an increase in levels of SCFAs in fecal batch culture (13–16) and in *in vivo* studies (18–20), thus showing prebiotic effects. The level of SCFA production and the fermentation rate were found to be dependent on XOS length and the presence of various substituents (15, 17). On the other hand, only one study has evaluated the fermentability of glucomannan and galactoglucomannan mixtures from pine wood by human fecal microbiota, reporting increased bifidobacterial numbers and higher acetate levels (21). In addition, Polari et al. showed that a galactoglucomannan mixture prepared from spruce sapwood promoted the growth of bifidobacteria in pure cultures (22).

Understanding how novel wood biomass-derived oligosaccharides impact the growth of commensal gut bacteria could lead to selective manipulation of the microbiota through their supplementation in diets, eventually promoting host health. Thus, the aim of the present study was to provide an insight into the potential prebiotic properties of AcGGM and AcAGX from Norwegian lignocellulosic biomass. *In vitro* single and batch cultures were used to determine their ability to stimulate the growth of beneficial human gut bacterial groups and to determine the profile of fermentative SCFAs corresponding to fecal human microbiota inocula.

## RESULTS AND DISCUSSION

### Preparation and characterization of the AcGGM and AcAGX samples.

The samples were prepared using methods similar to those described previously (11), except that the spruce wood was pretreated for 10 min at 200°C and then subjected to steam explosion treatment (23). Briefly, hemicelluloses from the pretreated spruce and birch wood samples were extracted by the use of warm water, and the extracts were filtered by bag filtering prior to ultrafiltration and nanofiltration. The retentate from the nanofiltration step was lyophilized, yielding AcGGM and AcAGX preparations from spruce and birch wood, respectively. Composition analysis showed that the molar ratio of mannose/glucose/galactose/acetyl units in the AcGGM preparation was 1:0.32:0.16:0.28, whereas the AcAGX preparation consisted of xylosyl units with glucuronic (uronic) acid, arabinose, and acetyl substituents in a molar ratio of 1:0.08:0.01:0.54 (Table 1). As expected, mannose (52.8%) and xylose (81.7%) were the most abundant monosaccharides in the AcGGM and AcAGX preparations, respectively. Arabinose (3.3%), uronic acids (2.4%), and xylose (16.2%) were also found in the AcGGM sample. These sugars likely resulted from the presence of a minor amount of xylan in the spruce wood, consistent with the results obtained in previous studies (24). Similarly, in the AcAGX sample, the levels of mannose (5.0%) and glucose (4.2%) indicated the presence of a minor amount of glucomannan in birch wood, according to data shown previously (25).

**TABLE 1:** Monosaccharide composition of AcGGM and AcAGX preparations obtained by HPAEC-PAD, HPLC, and colorimetric analyses^*a*^

Sample	Monosaccharide composition^*b*^ (mol/100 mol)					
	Ara	Gal	Glc	Xyl	Man	U^*c*^
AcGGM^*d*^	3.3	8.5	16.8	16.2	52.8	2.4
AcAGX^*e*^	0.8	1.9	4.2	81.7	5.0	6.3

a Ara, arabinose; Gal, galactose; Glc, glucose; Xyl, xylose; Man, mannose; U, uronic acid; Ac, acetyl group.

b Values (mol/100 mol [i.e., percentage value]) were calculated based on the total sugar molarity measured on each sample.

c The uronic acid (U) content is based on the adapted colorimetric assay developed by Scott (63) described in Materials and Methods.

d Man:Glc:Gal:Ac acetylation ratio (mol/mol) per mannose: 1:0.32:0.16:0.28. Please note that the degree of acetylation cannot be precisely evaluated since the fraction contains Xyl, which can be esterified by acetyl groups.

e Xyl:U:Ara:Ac acetylation ratio (mol/mol) per xylose: 1:0.08:0.01:0.54. Please note that the degree of acetylation cannot be precisely evaluated since the fraction contains Man, which can be esterified by acetyl groups.

**FIG 2:**
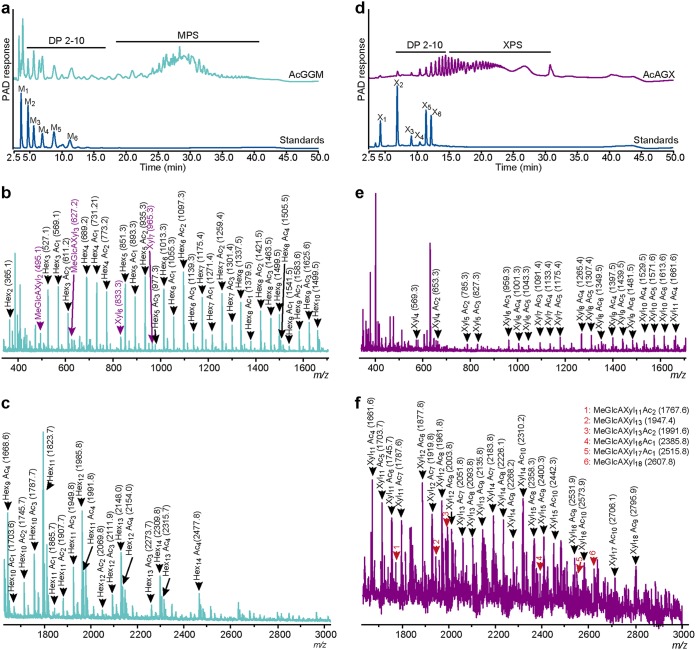
Characterization of the carbohydrate preparations. (a) HPAEC-PAD traces showing the manno-oligosaccharides (degree of polymerization [DP], between 2 and 10) and manno-polysaccharides (MPS) (DP, >10) present in the AcGGM preparation. The AcGGM sample was chromatographed with the following external standards: M_1_, mannose; M_2_, mannobiose; M_3_, mannotriose; M_4_, mannotetraose; M_5_, mannopentaose; M_6_, mannohexaose. (b and c) MALDI-TOF spectra of low-molecular-weight (b) to medium-molecular-weight (c) components present in the AcGGM preparation. Masses of the sodium adduct ion (Mass + Na^+^) are shown. (d) HPAEC-PAD traces showing the xylo-oligosaccharides (DP ranging from 2 to 10) and xylo-polysaccharides (XPS) (DP, >10) present in the AcAGX preparation. Samples were analyzed with the following external standards: X_1_, xylose; X_2_, xylobiose; X_3_, xylotriose; X_4_, xylotetraose; X_5_, xylopentaose; X_6_, xylohexaose. (e and f) MALDI-TOF spectra of low-molecular-weight (e) to medium-molecular-weight (f) components present in the AcAGX preparation. Spectra show sodium adducts (Mass + Na^+^). In panels b, c, d, and e, the following abbreviations are used: Ac, acetyl group; Hex, hexose; Me, methyl group; GlcA, glucuronic acid; Xyl, xylose.

To look for additional information on mass distribution and structural features, the two preparations were analyzed by high-performance anion-exchange chromatography (HPAEC) coupled with a pulsed amperometric detector (HPAEC-PAD) and matrix-assisted laser desorption–ionization time of flight mass spectrometry (MALDI-TOF) (Fig. 2). HPAEC-PAD analysis of the AcGGM preparation revealed the presence of β-MOS with DP of 2 to 10 and manno-polysaccharides (DP of ≥11) (Fig. 2a). The AcAGX preparation consisted predominantly of oligosaccharides with DP of 2 to 10 but also polymeric xylan (DP of ≥11) (Fig. 2d). Both preparations contained less than 1% monosaccharides. A deeper insight into the structure of the different components with low to medium molecular weight was obtained from the MALDI-TOF data. As shown in Fig. 2b and c, the AcGGM preparation contained a series of oligomers with DP ranging from 2 to 14 with single, double, triple, and quadruple acetylations. The degree of acetylation increased for higher-DP β-MOS; the most complex detectable components were DP 13 and DP 14 saccharides bearing four acetyl groups. The AcAGX preparation contained native and acetylated oligomers with DP ranging from 2 to 18 (Fig. 2e and f). The degree of acetylation increased in the higher-DP XOS, where DP 17 to 18 had up to 10 acetylations.

### Growth experiments with single bacterial cultures.

**FIG 3:**
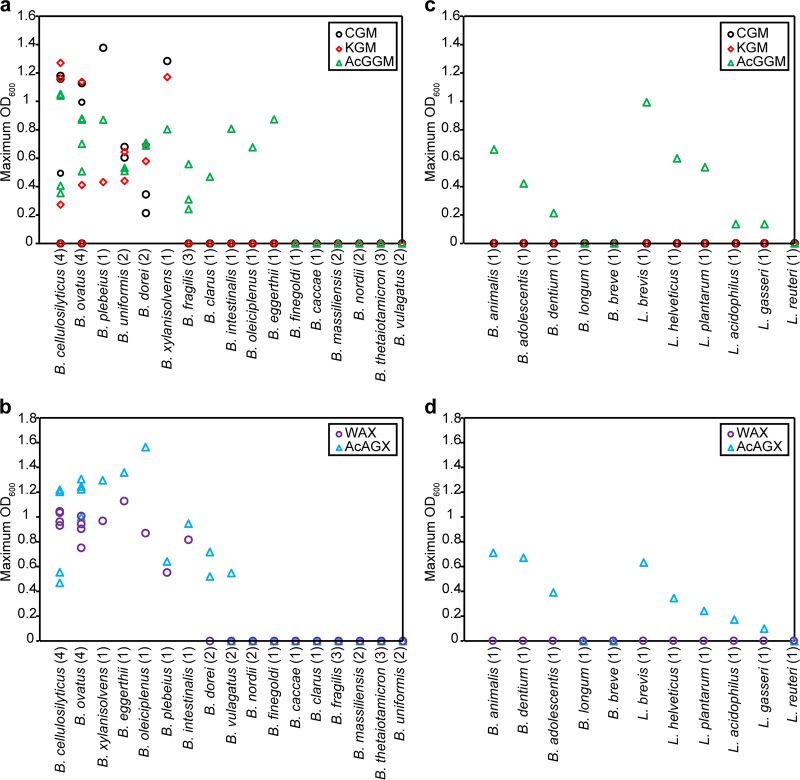
Utilization of xylans and β-mannans by selected *Bacteroides*, *Bifidobacterium*, and *Lactobacillus* members. (a and b) Bacteroides (number of strains shown in parentheses) were inoculated into MM containing individual β-mannans (CGM, carob galactomannan; KGM, konjac glucomannan; AcGGM, acetylated galactoglucomannan) (a) or xylans (WAX, wheat arabinoxylan; AcAGX, acetylated arabinoglucuronoxylan) (b) as the sole carbon source. (c and d) Bifidobacteria and lactobacilli (number of strains shown in parentheses) were cultivated in SDM supplemented with β-mannans (c) or xylans (d) as described above. Growth was measured as optical density at 600 nm (OD_600_). The figures show the maximum OD_600_ value for each culture corrected by the OD_600_ value of the baseline (carbohydrate-free control) at the same time. Data are averages from three biological replicates.

The ability of the gut microbes to degrade and metabolize β-MOS/β-mannans and XOS/xylans with various DP and substitutions has been reported for several numerically dominant and physiological relevant members of the gut microbiome, including members of the *Bifidobacterium*, *Lactobacillus*, and *Bacteroides* genera (22, 26, 27). In order to determine the fermentability of wood-derived AcGGM and AcAGX by these bacteria, independent *in vitro* culture experiments were conducted for a collection of 5 *Bifidobacterium* strains, 6 *Lactobacillus* strains, and 32 *Bacteroides* strains. Bacteria were cultured in either minimal medium (MM) (for *Bacteroides*) or semidefined medium (SDM) (for bifidobacteria and lactobacilli) supplemented with 0.5% (wt/vol) of either AcGGM (MM-AcGGM or SDM-AcGGM) or AcAGX (MM-AcAGX or SDM-AcAGX) separately, as the sole carbon source, and growth was evaluated as maximum optimal density at 600 nm (OD_600_). Commercial polymeric β-mannans (carob glucomannan [CGM] and konjac galactomannan [KGM]) and xylans (wheat arabinoxylan [WAX]) were used as controls. All the bacteria displayed growth on MM or SDM supplemented with 0.5% (wt/vol) glucose (see Table S1 in the supplemental material). Bacteroides cellulosilyticus, B. ovatus, B. plebeius, B. uniformis, B. dorei, and B. xylanisolvens all grew in the presence of AcGGM, whereas they displayed various levels of the ability to metabolize either CGM or KGM (Fig. 3a). B. fragilis, B. clarus, B. intestinalis, B. oleiciplenus, and B. eggerthii achieved considerable growth on AcGGM but failed to grow in the presence of both CGM and KGM (Fig. 3a). These data suggest that these strains might be able to utilize the low-molecular-weight β-MOS present in the AcGGM mixture but cannot metabolize the high-molecular-weight β-mannans CGM and KGM. AcAGX supported the growth of B. cellulosilyticus, B. ovatus, B. xylanisolvens, B. eggerthii, B. oleiciplenus, B. plebeius, B. intestinalis, B. dorei, and B. vulgatus (Fig. 3b). Under these conditions, most of the bacteria reached higher maximum OD_600_ levels than those observed on WAX. Overall, several isolates did not show growth in MM-AcGGM or MM-AcAGX, consistent with the fact that the efficient utilization of β-mannans and xylans is not universal in the gut resident *Bacteroides* (28).

10.1128/mSphere.00554-18.6 TABLE S1 Bacterial strains used in this study. Download Table S1, PDF file, 0.2 MB. Copyright © 2019 La Rosa et al. 2019 La Rosa et al. This content is distributed under the terms of the Creative Commons Attribution 4.0 International license.

Bifidobacterium adolescentis, B. animalis subsp. *lactis* Bl-04, and B. dentium were capable of fermenting AcGGM and AcAGX, while all strains failed to grow on the pure polymeric substrates CGM, KGM, and WAX (Fig. 3c and d). These results are in agreement with a previous study showing that only a few of the *Bifidobacterium* strains isolated from the human gut were capable of growth on high-molecular-weight XOS and MOS (22, 29).

Lactobacilli, in contrast, do not metabolize XOS and MOS significantly (30), with the exception of Lactobacillus brevis (31). However, they can utilize linear short-chain oligosaccharides (DP of 2 to 3), as exemplified by the uptake of mannobiose and mannotriose by L. plantarum WCFS1 (see Fig. S4 in the supplemental material). This is in agreement with our results, where L. brevis grew well on SDM-AcGGM and SDM-AcAGX whereas the other strains displayed limited growth on these substrates (Fig. 3c and d). None of the lactobacilli displayed growth on CGM, KGM, and WAX (Fig. 3c and d).

### Identification of gene clusters for metabolism of β-MOS/β-mannans and XOS/xylans.

Genomic analyses revealed the presence of putative gene clusters that may sustain growth on β-MOS/β-mannans and XOS/xylans in specific *Bacteroides, Bifidobacterium*, and *Lactobacillus* strains (Fig. S1, S2, and S3). In *Bacteroides*, the annotated polysaccharide utilization loci (PUL) for β-mannan (PUL-Mannan) and xylan (PUL-Xyl) degradation contain a classical SusC/SucD sensor/transcriptional regulator and one or more genes encoding predicted CAZymes, including β-mannanases (glycoside hydrolase 26 [GH26], GH5), β-glucosidases (GH2, GH3), α-galactosidase (GH36), carbohydrate esterases (CE1-6, CE7, CE9), xylanases (GH10), α-arabinofuranosidase (GH43), and α-glucuronosidase (GH115) (25–27) (Fig. S1 and S2). Xylan degradation systems have been previously described and characterized at the enzyme level in B. ovatus, B. xylanisolvens, B. intestinalis, and B. cellulosilyticus (32–35). In addition, we detected large PUL-Xyls in the genome of B. eggerthii, B. oleiciplenus, and B. plebeius and smaller ones in the genome of B. vulgatus and B. dorei (Fig. S1). The galactomannan utilization locus in B. ovatus has been described previously, and the biochemical properties of two GH26 β-mannanases, *Bo*Man26A and *Bo*Man26B, and a GH36 α-galactosidase (*Bo*Gal36A) have been characterized (36, 37). A homologous PUL was detected in B. xylanisolvens strains capable of growing on galactomannan (37). McNulty et al. revealed the presence of putative GH26 β-mannanase genes in the genome of B. cellulosilyticus WH2 (33); these genes are located in three putative PULs, with one similar to the PUL involved in galactomannan degradation in B. ovatus (Fig. S2). We observed a PUL-Mannan similar to that described for B. fragilis (38) in the genome of B. intestinalis and B. eggerthii. B. uniformis, B. dorei, B. clarus, and B. oleiciplenus harbored PUL-Mannans with previously undescribed gene configurations (Fig. S2). Notably, the large variations in gene content and organization observed when comparing the putative xylan and β-mannan PULs from different *Bacteroides* underscore how its members may have evolved diverse strategies for the exploitation of these hemicelluloses.

10.1128/mSphere.00554-18.1 FIG S1 Organization of the xylan utilization loci based on the annotated *Bacteroides* genomes. Genes are depicted based on the function of their products as follows: glycoside hydrolase (GH) genes in red, carbohydrate esterase (CE) in blue, *sus*C-like genes in purple, *sus*D-like genes in orange, and hybrid two-component system (HTCS) genes in green. Genes encoding proteins of unknown function are shown in white. An asterisk indicates that the PUL has been already described in/biochemical data are available from previous studies. This applies to B. cellulosilyticus (33), B. ovatus and B. xylanisolvens (32, 34) and B. intestinalis (35). The PULs for which biochemical data are available are highlighted in yellow. Download FIG S1, TIF file, 0.4 MB. Copyright © 2019 La Rosa et al. 2019 La Rosa et al. This content is distributed under the terms of the Creative Commons Attribution 4.0 International license.

10.1128/mSphere.00554-18.2 FIG S2 Organization of the β-mannan utilization loci based on the annotated *Bacteroides* genomes. Genes are depicted according to the function of their products as follows: glycoside hydrolase (GH) genes in red, carbohydrate esterase (CE) genes in blue, *sus*C-like genes in purple, *sus*D-like genes in orange, hybrid two-component system (HTCS) genes in green, and transcriptional regulator (TR) genes in yellow. Genes encoding proteins of unknown function are shown in white. An asterisk indicates that the PUL has been already described in/biochemical data are available from previous studies. This applies to B. cellulosilyticus (33), B. ovatus and B. xylanisolvens (36, 37), and B. fragilis (38). The PULs for which biochemical data are available are highlighted in yellow. Download FIG S2, TIF file, 0.3 MB. Copyright © 2019 La Rosa et al. 2019 La Rosa et al. This content is distributed under the terms of the Creative Commons Attribution 4.0 International license.

10.1128/mSphere.00554-18.3 FIG S3 Gene clusters for XOS and β-MOS utilization in bifidobacteria and lactobacilli. (a) Organization of the gene cluster for arabinoxylo-oligosaccharide utilization detected in the tested *Bifidobacterium* strains. The organization of these genes was described previously (40). (b) Schematic view of the gene cluster potentially involved in the utilization of linear short-chain β-MOS and XOS in lactobacilli. The gene cluster in L. gasseri ATCC 33323 was described in reference 45, while the gene-encoded proteins involved in xylan degradation in L. brevis ATCC 14869 were characterized in reference 31. In both panel a and panel b, genes are depicted based on the function of their products as follows: glycoside hydrolase (GH) genes in red, carbohydrate esterase (CE) genes in blue, ABC transporter genes in orange (AXBP, arabino-xylan binding protein; Perm, permease), and transcriptional regulator (TR) genes in yellow. Genes encoding non-CAZymes are shown in light green. HPr, kinase/phosphorylase. Download FIG S3, PDF file, 0.8 MB. Copyright © 2019 La Rosa et al. 2019 La Rosa et al. This content is distributed under the terms of the Creative Commons Attribution 4.0 International license.

10.1128/mSphere.00554-18.4 FIG S4 Utilization of manno-oligosaccharides by Lactobacillus plantarum WCFS1. (a) Growth curves of L. plantarum WCFS1 on minimal medium (MM) supplemented with 0.5% glucose (blue), polymeric carob galactomannan (pink), GH26-treated carob (orange), or GH36/GH26-treated carob (red) or without supplementation of any carbon source (dark red). Growth was measured as the optical density at 600 nm (OD_600_). (b) pH measurements before (light blue) and after (purple) overnight culturing of L. plantarum on the same substrates as described for panel a. In both panel a and panel b, data are representative of three independent experiments. (c) HPAEC-PAD analysis of MM supplemented with untreated carob, GH26-treated carob, and GH36/GH26-treated carob before and after fermentation with L. plantarum. Numbers 1 to 6 on the uppermost chromatogram indicate the positions of mannose (1), mannobiose (2), mannotriose (3), mannotetraose (4), mannopentaose (5), and mannohexaose (6). The asterisk indicates the galactose peak. Download FIG S4, TIF file, 0.3 MB. Copyright © 2019 La Rosa et al. 2019 La Rosa et al. This content is distributed under the terms of the Creative Commons Attribution 4.0 International license.

In contrast to *Bacteroides*, bifidobacteria lack extracellular endo-xylanases (GH10), thus exhibiting no hydrolytic capabilities with respect to polymeric xylans (39). However, previous studies have shown that several members of the *Bifidobacterium* genus are able to grow on XOS and that XOS uptake and metabolism rely on the presence of an ATP-binding cassette (ABC) transporter and cytoplasmic enzymes that process these to their monosaccharide components (40) (Fig. S3a). B. animalis subsp. *lactis* Bl-04 possesses an arabinoxylo-oligosaccharide (AXOS) utilization locus (Fig. S3a) consisting of a LacI transcriptional regulator, xylosidases (GH8, GH43, and GH120), arabinofuranosidases (GH43), and acetyl esterases for the removal of arabinosyl and acetyl substituents, as well as an ABC transporter (40, 41). Consistent with the fact that this locus is conserved across different XOS-utilizing *Bifidobacterium* species, it was found in the genome of the B. adolescentis and B. dentium (40) (Fig. S3a). Some *Bifidobacterium* strains, including B. adolescentis, can ferment GGM, KGM, and glucomannan oligosaccharides (22, 42); nevertheless, the β-mannan degradation system in bifidobacteria remains largely underexplored. A study reported by Kulcinskaja et al. (43) characterized a multimodular B. adolescentis β-mannanase (BaMan26A), although the ability of this bacterium to hydrolyze all of the potential linkages found in β-mannan has not been confirmed. BaMan26A homologs were found in the genome of β-mannan-utilizing B. animalis subsp. *lactis* Bl-04 and B. dentium (Table S1).

Carbohydrates are mainly imported by phosphoenolpyruvate (PEP):carbohydrate phosphotransferase system (PTS) transporters in lactobacilli (44). These systems consist of cytoplasmic components (enzyme E1 and histidine-phosphorylatable protein HPr) that lack sugar specificity and of membrane-associated enzymes (enzymes E2A, E2B, E2C, and sometimes E2D) that transfer a phosphate group from PEP to the incoming sugar, preventing it from diffusing back out of the cell. Glycans are then hydrolyzed by specific phospho-β-glucosidases (GH1) into the glycolytic precursors glucose-6-phosphate and glucose. These glycan utilization loci are conserved in the genome of L. plantarum WCFS1, L. helveticus, L. acidophilus, and L. gasseri (45) (Fig. S3b). This system would allow the bacteria to transport and metabolize short-chain undecorated XOS and β-MOS, whereas larger polysaccharides could not be processed. Indeed, polymeric CGM did not support growth of L. plantarum WCFS1, whereas the bacterium was able to grow and take up β-MOS derived from GH36/GH26 hydrolysis of CGM (Fig. S4). As an exception, L. brevis is capable of fermenting arabinoxylan-oligosaccharide (AXOS) through the action of arabinofuranosidases, including a GH43 and two GH51s (31, 46). Overall, consistent with the absence of metabolism involving β-MOS/β-mannans and XOS/xylans, no related gene clusters were found in the genome of the *Bacteroides, Bifidobacterium*, and *Lactobacillus* strains lacking the ability to grow on these hemicelluloses *in vitro* (Table S1).

### *In vitro* fermentation of AcGGM and AcAGX.

**FIG 4:**
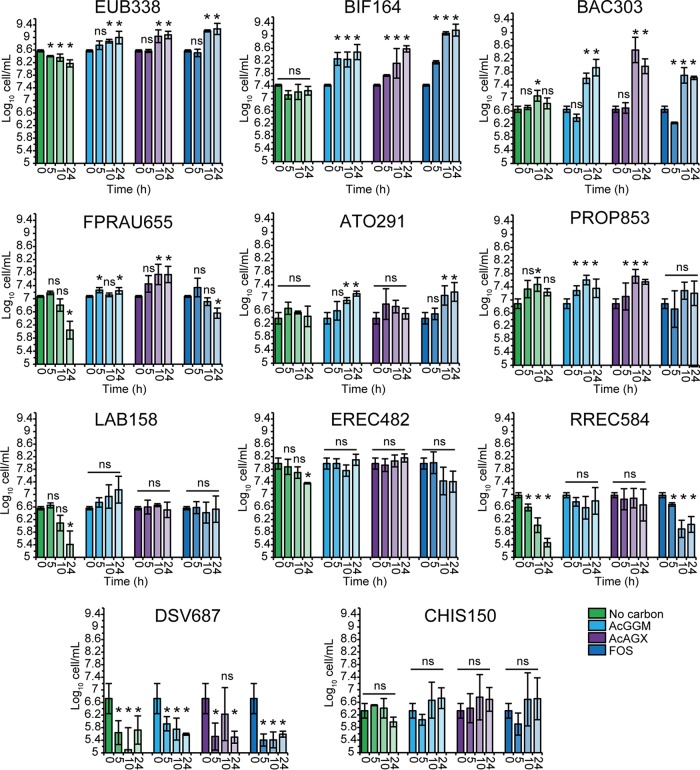
FISH-FCM analysis. Bacterial enumeration was performed using FISH-FCM following batch culture fermentation with no carbon source (green) or with AcGGM (turquoise), AcAGX (magenta), and FOS (blue). Cultures were inoculated with healthy human fecal microbiota, and samples were taken at 0, 5, 10, and 24 h. Histograms depict means of data from three biological replicates ± standard deviations. An asterisk (*) indicates a significant difference (*P* < 0.05) compared with the value at 0 h; ns, nonsignificant (*P* ≥ 0.05). EUB338, total bacteria; BIF164, *Bifidobacterium* spp.; BAC303, *Bacteroides*-*Prevotella* spp.; FPRAU655, Faecalibacterium prausnitzii cluster; ATO291, *Atopobium* cluster; PROP853, clostridial cluster IX; LAB158, *Lactobacillus*-*Leuconostoc*-*Enterococcus* spp.; EREC482, clostridial clusters XIVa and XIVb; RREC584, *Roseburia* spp.; DSV687, *Desulfovibrionales* and *Desulfuromonales*; CHIS150; Clostridium histolyticum group.

Batch pH-controlled fermentations were set up to investigate the prebiotic potential of the AcGGM and AcAGX mixtures. Samples were taken at designated time intervals for enumeration of physiologically relevant bacterial groups by fluorescent *in situ* hybridization (FISH) coupled with flow cytometry (FISH-FCM). For comparisons, commercial FOS was used as a positive control due to its established prebiotic properties (6). Results presented in Fig. 4 show an increase of the total bacterial population (enumerated using the EUB338 probe) after 10 h of fermentation in response to both substrates and FOS. In the absence of any carbon source (negative control), the total bacterial numbers decreased during fermentation, thus confirming the suitability of AcGGM and AcAGX as substrates for the metabolism of the human fecal microbiota. Comparisons of both substrates with FOS revealed that the total bacterial populations at the end of the fermentation were not significantly different (*P > *0.05). Counts of bifidobacteria (probe BIF164) increased significantly after 5 h of fermentation in response to the positive control and to both AcGGM and AcAGX. At 24 h of fermentation, the highest increase (of 1.7 ± 0.1 log_10_ cells/ml) was observed in the samples supplemented with FOS, followed by increases of 1.1 ± 0.2 and 1.1 ± 0.3 log_10_ cells/ml in AcGGM and AcAGX samples, respectively. Previous studies indicated that an increase of 0.5 to 1.0 log_10_ cells/ml in the population of bifidobacteria elicits a major shift in the gut microbiota toward a healthier composition in infants and adults (47, 48). Thus, both AcGGM and AcAGX can be considered bifidogenic under the conditions tested. Fermentation of AcGGM and AcAGX for 24 h increased the *Bacteroides*-*Prevotella* (probe BAC303) populations significantly (by 1.3 ± 0.2 log_10_ cells/ml), while FOS increased the population by 0.9 ± 0.1 log_10_ cells/ml. These results are in agreement with those obtained for low-molecular-weight XOS from oil palm empty fruit bunches and corn cobs (13, 15). Similar results were also reported with wood-derived β-MOS (21). Previous studies demonstrated that bifidobacteria are equipped with efficient machinery for utilization of XOS (40); thus, the increase in the population could be attributed to the fermentation of the low-molecular-weight undecorated and acetyl-decorated XOS which have been shown to be present in the AcAGX mixture (Fig. 2e and f). Alternatively, it is likely that the *Bacteroides* serve as primary degraders of the high-molecular-weight carbohydrates into shorter molecules; bifidobacteria can eventually utilize these degradation products thanks to their cross-feeding behavior (39).

While the populations of butyrogenic bacteria of the Faecalibacterium prausnitzii (probe FPRAU655) cluster and of propionate-producing bacteria belonging to clostridial cluster IX (probe PROP853) declined or remained stable throughout FOS fermentation, overall significant increases were detected with AcGGM (0.3 ± 0.01 log_10_ cells/ml for FPRAU655 and 0.6 ± 0.2 log_10_ cells/ml for PROP853) and AcAGX (0.8 ± 0.3 log_10_ cells/ml for FPRAU655 and 0.7 ± 0.1 log_10_ cells/ml for PROP853) between 0 and 24 h. In pure culture experiments, F. prausnitzii DSM 17677 was not able to degrade CGM, KGM, or arabinoxylans from oat and wheat to any extent (27). However, the increased numbers of F. prausnitzii cells in the batch culture experiment performed with AcGGM and AcAGX can be explained by the presence of strains equipped with machinery for utilization of these hemicelluloses. Indeed, we identified a cluster encoding enzymes involved in β-mannan metabolism in two F. prausnitzii strains whose genome were sequenced (Fig. S5). The cluster lacks a GH26 endomannanase, as would be required to hydrolyze the β-mannan backbone into smaller oligosaccharides, but it may allow growth of these F. prausnitzii strains through cross-feeding on β-MOS released by other colonic microorganisms. F. prausnitzii strain 2789STDY5834930, isolated in the United Kingdom from a fecal sample, possesses two β-xylosidases/arabinosidases (ERS852542_01151/01152) that might support the growth on AXOS. It is likely that similar strains present in the fecal samples that we employed in this study are able to hydrolyze and ferment the XOS present in the AcAGX preparation. Aside from being a ubiquitous primary source of butyrate in the colon, F. prausnitzii is thought to regulate gut homeostasis and to have anti-inflammatory properties (49). Thus, stimulation of F. prausnitzii growth through AcGGM and AcAGX treatments could result in an overall beneficial effect on host health.

10.1128/mSphere.00554-18.5 FIG S5 Schematic of the utilization loci of β-manno-oligosaccharides in F. prausnitzii SL3/3 and M21/2. Genes are depicted based on the function of their products as follows: glycoside hydrolase (GH) genes in red, carbohydrate esterase (CE) genes in blue, ABC transporter genes in orange (MnBP, manno-oligosaccharide binding protein; MPP, manno-oligosaccharide permease protein), and transcriptional regulator (TR) genes in yellow. Genes encoding non-CAZymes are shown in light green. In F. prausnitzii SL3/3, the two gene clusters correspond to the loci FPR_17170-17320 and FPR_09740-09750. In F. prausnitzii M21/2, the two gene clusters correspond to the loci FAEPRAM212_01132-01147 and FAEPRAM212_01007-01008. Download FIG S5, TIF file, 2.5 MB. Copyright © 2019 La Rosa et al. 2019 La Rosa et al. This content is distributed under the terms of the Creative Commons Attribution 4.0 International license.

The counts of another member of the *Actinobacteria* group, i.e., the *Atopobium* cluster (probe ATO291), increased after 24 h of growth with AcGGM (0.8 ± 0.3 log_10_ cells/ml) and FOS (0.9 ± 0.1 log_10_ cells/ml), whereas AcAGX did not enhance its growth. These findings are in line with previous studies reporting that FOS promoted the growth of *Atopobium*, which, on the other hand, was unaffected by high-molecular-weight XOS or AXOS (15, 50). To date, no study has investigated the β-mannan fermentation capabilities of the *Atopobium* cluster; thus, it remains unclear whether the increase in this population seen with the AcGGM mixture is supported by the activity of mannolytic *Atopobium* strains.

No significant changes were detected at any time point in other bacterial populations, namely, *Lactobacillus* spp. (probe LAB158), Clostridium coccoides-Eubacterium rectale genus (probe EREC482), and the Clostridium histolyticum group (probe CHIS150). AcGGM and AcAGX supported a stable *Roseburia* sp. population throughout the fermentation, whereas a significant decrease was seen in the cultures supplemented with FOS after 5 h. Also, an overall decrease in numbers of the sulfate-reducing *Desulfovibrio* spp. was observed with all substrates tested.

Although the total number of bacteria decreased during fermentation in the absence of any carbon source, we observed an increase in the populations of clostridium cluster IX and *Bacteroides* after 10 h of fermentation and constant numbers of bifidobacteria and the *Atopobium* cluster bacteria over time (Fig. 4). Growth of the *Bacteroides* members is consistent with their ability to utilize peptide and yeast α-mannan/β-glucans, deriving from the peptone and yeast extract components of the basal medium as previously shown (28, 51).

### Organic acid analysis.

Table 2 summarizes the amounts of the SCFAs acetate, propionate, and butyrate as measured by the use of a gas chromatograph analyzer equipped with a flame ionization detector (GC-FID) in the fermentation supernatants of the batch cultures over time. Total organic acid concentrations increased throughout the fermentation, and the increases were more pronounced in the cultures supplemented with the carbohydrates than in the negative-control samples (*P < *0.05). Acetate was the dominant SCFA produced (65% to 75% of the total) for all substrates tested, with significant differences seen at all time points. The highest concentration was achieved in the cultures containing AcAGX, followed by those containing FOS and AcGGM. The increase in acetate levels is consistent with the higher abundance of bifidobacteria detected by FISH-FCM. Previous studies (14, 15) have shown that fermentation of XOS resulted in a higher concentration of acetate, which is in agreement with the present findings. Acetate is considered the primary SCFA in colonic fermentation and is used for *de novo* lipogenesis once it enters the systemic circulation. In addition, the increase in acetate could result in a more acidic colonic pH, thus inhibiting the proliferation of pathogenic bacteria, which are generally sensitive to low pH values (52).

**TABLE 2:** Concentrations of acetate, propionate, and butyrate during fermentation with different carbohydrates or no carbon source^*a*^

Organic acid	Time (h)	Concn (mM)			
		No carbon	AcGGM	AcAGX	FOS
Total	0	0.00 ± 0.00	0.00 ± 0.00	0.00 ± 0.00	0.00 ± 0.00
	5	0.00 ± 0.00	25.08 ± 7.06*	31.42 ± 2.57*	23.6 ± 23.08^#^
	10	4.15 ± 3.32	45.39 ± 17.99*	45.63 ± 14.87*	51.33 ± 7.74*
	24	19.63 ± 1.68	100.53 ± 21.1*	107.89 ± 14.59*	100.14 ± 30.82*

Acetate (A)	0	0.00 ± 0.00	0.00 ± 0.00	0.00 ± 0.00	0.00 ± 0.00
	5	0.00 ± 0.00	24.40 ± 6.43*	29.45 ± 1.77*	22.25 ± 21.43^#^
	10	3.22 ± 2.51	40.09 ± 12.96*	37.05 ± 10.47*	47.20 ± 3.47*
	24	14.79 ± 0.81	65.60 ± 11.53*	76.83 ± 8.20*	75.62 ± 14.37*

Propionate (P)	0	0.00 ± 0.00	0.00 ± 0.00	0.00 ± 0.00	0.00 ± 0.00
	5	0.00 ± 0.00	0.63 ± 0.55*	1.77 ± 0.45*	1.03 ± 1.79^#^
	10	0.93 ± 0.81	2.17 ± 1.64^#^	7.28 ± 3.83*	1.25 ± 1.51^#^
	24	3.01 ± 0.53	12.78 ± 4.92*	23.76 ± 4.13*	9.18 ± 2.17*

Butyrate (B)	0	0.00 ± 0.00	0.00 ± 0.00	0.00 ± 0.00	0.00 ± 0.00
	5	0.00 ± 0.00	0.05 ± 0.08^#^	0.20 ± 0.35^#^	0.32 ± 0.56^#^
	10	0.00 ± 0.00	3.13 ± 3.39^#^	1.30 ± 0.57*	2.88 ± 2.76^#^
	24	1.83 ± 0.34	22.15 ± 4.65*	7.3 ± 2.26*	15.34 ± 14.28^#^
					
A:P:B ratio	0	0:0:0	0:0:0	0:0:0	0:0:0
	5	0:0:0	1.0.025:0.002	1:0.06:0.007	1:0.05:0.014
	10	1:0.28:0	1:0.05:0.08	1:0.2:0.035	1:0.03:0.06
	24	1:0.2:0.12	1:0.19:0.34	1:0.31:0.095	1:0.12:0.2

a Values represent the means ± standard deviations of results from three biological replicates. Asterisks (*) indicate significant differences (*P *<* *0.05) from the baseline value (no carbon) at the same time point. Nonsignificant differences (*P *≥* *0.05) are indicated with a hash mark (^#^).

The production profile of propionate was similar to that of acetate, although it reached lower levels. This is in accordance with the significant enhancement in the *Bacteroides*-*Prevotella* and clostridial cluster IX populations, as these bacteria are known to be propionate producers (53). The propionate levels of AcGGM at 24 h were comparable to those measured for FOS; however, fermentation of AcAGX generated more propionate (1:0.31:0.095 mM [acetate/propionate/butyrate] ratio) than fermentation of AcGGM and FOS (Table 2). Propionic acid production has been reported to result in beneficial health effects on the host, including appetite regulation, lower levels of glucose-induced insulin secretion, and antiproliferative effects on liver cancer cells (54, 55). Moreover, while acetate acts as a precursor for biosynthesis of blood lipids, propionate is able to inhibit this process. Thus, a low acetate/propionate ratio helps in regulating levels of blood lipids and potentially reducing cardiovascular disease risk (54).

The wood-derived substrates tested generated a major change in the level of butyrate production (*P < *0.05). This is in good agreement with the results showing an increased abundance of F. prausnitzii, which is a major butyrate producer, in the samples supplemented with AcGGM and AcAGX. Interestingly, fermentation of AcGGM generated more butyrate (1:0.19:0.34 mM [acetate/propionate/butyrate] ratio) than fermentation of AcAGX and FOS at 24 h, indicating that that substrate promotes butyrogenic fermentation in particular (Table 2). The ability of FOS to promote butyrate synthesis has been reported previously, and it has been linked to the conversion of acetate into butyrate by the gut microbiota (15, 56). The production of butyrate by commensal bacteria has received much attention for its health-promoting functions (57). It serves as the main energy source for colonocytes (58) and exhibits anticarcinogenic, anti-inflammatory, and barrier-protective properties in the distal gut (59–61).

Fermentation of the negative control resulted in a limited but still significant increase of acetate, propionate and butyrate levels after 10 h of fermentation (Table 2). Production of acetate and propionate could be linked to the increases in the levels of the *Bacteroides* and clostridial cluster IX populations (53). The gut microbiota is capable of converting some of the acetate into butyrate that thus does not originate from the butyrate-producing capacity in the fecal slurry (62).

### Conclusions.

Our results prove that AcGGM and AcAGX, which consist of oligosaccharides and polysaccharides with different DP and structure, can influence the composition of the gut microbiota. Both preparations displayed *in vitro* prebiotic activity, in terms of increasing the number of bifidobacteria, besides stimulating the growth of other beneficial bacteria, including the *Bacteroides*-*Prevotella*, clostridial cluster IX, and F. prausnitzii groups. Metabolite production correlated well with changes in bacterial populations; acetate was the prevalent SCFA released, followed by butyrate for AcGGM and propionate for AcAGX. The total levels of SCFAs produced by fermentation of AcGGM and AcAGX were comparable with those seen with the established prebiotic FOS.

Although further *in vivo* investigations should be conducted to ultimately confirm the beneficial health effect for the host, AcGGM and AcAGX from lignocellulosic biomass can be considered new potential prebiotics for human consumption.

## MATERIALS AND METHODS

### Substrates and standards.

Xylan and β-mannan were isolated from multiple batches of steam-exploded Norwegian birch and spruce wood, respectively, as described previously (11). Briefly, wood sawdust was pretreated by steam explosion (10-min residence time at 200°C with a solid-to-liquid ratio of 1:1 [wt/wt]). The hemicellulose released was extracted by the use of hot water, and the resulting soluble material was subjected to ultrafiltration using 1-kDa- and 5-kDa-molecular-weight-cutoff membranes (Alfa Laval, Sweden) for xylan and β-mannan, respectively. The retentates from the ultrafiltration were concentrated on a nanofiltration membrane (TriSep 2540-XN45-TSF membrane; Lenntech, The Netherlands). Ultrafiltration and nanofiltration were conducted using UF/NF model G filtration (Gea, Denmark). The nanofiltration retentate was lyophilized on an Alpha 2-4 LD Plus freeze-dryer (Christ, Germany), yielding further two preparations named acetylated galactoglucomannan (AcGGM) and acetylated arabinoglucuronoxylan (AcAGX). Fructo-oligosaccharides (FOS; Actilight) were obtained from Tereos (Lille, France). Manno- and xylo-oligosaccharides (DP, 2 to 6), konjac glucomannan (KGM), carob galactomannan (CGM), and wheat arabinoxylan (WAX) were purchased from Megazyme International (Wicklow, Ireland). Acetic, propionic, and butyric acid standards were of analytical grade and were obtained from Sigma-Aldrich (St. Louis, MO, USA).

### Sugar composition analysis.

The neutral sugar composition of AcGGM and AcAGX was analyzed using HPAEC (Dionex ICS-3000) (CarboPac PA1 columns [2 × 50 mm and 2 × 250 mm]; 30°C) coupled with a PAD detector (HPAEC-PAD). Samples (5 mg/ml) were heated at 121°C for 1 h with 4% H_2_SO_4_ to induce hydrolysis. Hydrolysates were diluted with 15 mM NaOH to obtain an optimal range of concentrations for the quantification. Neutral sugars were eluted with an isocratic concentration of 1 mM NaOH at a flow rate of 0.25 ml/min for 35 min. The uronic acid quantification was adapted from reference 63. Hydrolysis was initiated in silicate tubes with a mixture of 0.3 ml of polysaccharides (1 mg/ml) and 0.3 mg sodium boric acid solution (2% boric acid and 3% NaCl dissolved in water). Samples were incubated at 70°C for 40 min with 5 ml of concentrated sulfuric acid. The hydrolysates were cooled for 20 min. The colorimetric reaction was performed with the addition of 0.2 ml dimethylphenol (0.1% [wt/vol] in glacial acetic acid) at room temperature (RT) for 15 min and kept on ice until the absorbance was read. The total uronic concentration was proportional to the absorbance difference at wavelengths of 450 nm and 400 nm using glucuronic acid as a standard. The quantification of acetic acid was initiated with the saponification of the acetyl groups; this was done by incubating the carbohydrates (10 mg/ml) overnight at 4°C with 0.1 M NaOH to release bound acetyl groups. The analysis was performed using a Dionex Ultimate 3000 high-performance liquid chromatography (HPLC) system with 30 min of isocratic elution (5 mM H_2_SO_4_) on a Rezex ROA-organic acid-H+ column (7.8 × 300 mm) coupled to a Carbo-H4 security guard cartridge (3.0-mm internal diameter). Acetic acid was detected at the wavelength of 210 nm.

### Analysis of sugar monosaccharides and oligosaccharides by HPAEC-PAD.

Samples were analyzed by HPAEC-PAD using a Dionex ICS-3000 chromatographic system operated using Chromeleon software version 7 (Dionex, Thermo Scientific). The system was equipped with a CarboPac PA1 analytical column (Dionex, Thermo Scientific) (2 × 250 mm) in combination with a CarboPac PA1 guard column (2 × 50 mm), and it was run at a flow rate of 0.25 ml/min. The elution conditions for the analysis of manno-oligosaccharides and manno-polysaccharides were 0 to 9 min 0.1 M NaOH; 9 to 35 min 0.1 M NaOH with a 0 to 0.3 M sodium acetate (NaOAc) gradient; 35 to 40 min 0.1 M NaOH with 0.3 M NaOAc; and 40 to 50 min 0.1 M NaOH. The elution conditions for the xylo-oligosaccharides and xylo-polysaccharides were 0 to 10 min 0.1 M NaOH with a 0 to 0.1 M NaOAc gradient; 10 to 35 min 0.1 M NaOH with a 0.1 to 0.3 M NaOAc gradient; 35 to 40 min 0.1 M NaOH with a 0.3 to 1 M NaOAc gradient; and 40 to 50 min 0.1 M NaOH. Commercial manno-oligosaccharides and xylo-oligosaccharides (DP, 2 to 6) from Megazyme were used as standards.

### MALDI-TOF.

MALDI-TOF analyses were conducted using an Ultraflex MALDI-TOF/TOF instrument (Bruker Daltonics, Germany) equipped with a 337-nm-wavelength nitrogen laser. All measurements were performed in positive mode. Data were collected from 100 laser shots using the lowest energy level necessary to obtain sufficient spectral intensity. The mass spectrometer was calibrated with a mixture of manno-oligosaccharides or xylo-oligosaccharides (DP, 2 to 6) obtained from Megazyme. For sample preparation, 1 μl of sample solution was mixed with 2 μl of matrix (9% 2,5-dihydroxybenzoic acid [DHB]–30% acetonitrile [vol/vol]), directly applied on a MTP 384 target plate (Bruker Daltonics, Germany), and dried under a stream of warm air.

### Bacterial strains and growth assays.

The bacterial strains used in this study are listed in Table S1 in the supplemental material. Most of the bacterial strains were of human origin and were selected to represent the genetic diversity of the *Bacteroides*, *Lactobacillus*, and *Bifidobacterium* genera. The years of isolation of the selected strains spanned 1960 to >2000, and the strains originated in most parts of the world, including the United States, Europe, and Japan.

*Bacteroides* species were routinely cultured in a custom chopped meat (CM) medium (64) overnight at 37°C in an anaerobic chamber (Coy Lab Products, MI) under an 85% N_2_, 5% CO_2_, and 10% H_2_ atmosphere. Bifidobacteria and lactobacilli were grown in de Man-Rogosa-Sharpe medium (MRS) (Oxoid, United Kingdom) overnight at 37°C in an anaerobic cabinet (Whitley A95 workstation; Don Whitley, United Kingdom) when appropriate. To evaluate the bacterial growth in pure cultures, *Bacteroides* cells were pregrown overnight in minimal medium (MM) (65) supplemented with a monosaccharide mixture (glucose, mannose, xylose, galactose, fructose, and *N*-acetylglucosamine from Sigma-Aldrich). Bifidobacteria and lactobacilli were grown overnight in semidefined medium (66) supplemented with 0.5 mg/ml glucose. Aliquots (1 ml) of bacterial cultures were centrifuged to pellet the cells, which were then washed with 1 ml of the appropriate medium without carbon source, prior to exposure to each single carbohydrate (5 mg/ml). The growth experiments performed with *Bacteroides* were conducted in 200-µl cultures in 96-well microtiter plates. Growth was assessed by measuring the optical density (absorbance) at 600 nm (OD_600_) at 15-min intervals for 24 to 96 h by using either a Powerwave HT absorbance reader coupled with a Biostack plate handling device (Biotek Instruments, Winooski, VT) or a Spectrostar Nano microplate reader (BMG Labtech, USA). The growth experiments performed with bifidobacteria and lactobacilli were conducted in 2-ml culture volumes, and growth was assessed by measuring the optical density (absorbance) at 600 nm (OD_600_) at 8-h intervals for 24 to 96 h. Each species was tested in triplicate for each carbohydrate.

### *In vitro* batch fermentation.

The prebiotic potential of wood-based AcAGX and AcGGM was evaluated using an *in vitro* batch culture fermentation system inoculated with human fecal inocula. Briefly, sterile stirred batch culture fermentation vessels (20-ml working volume) were filled with 17 ml of sterile basal medium and gassed overnight with oxygen-free nitrogen (15 ml/min) to obtain anaerobic conditions. The basal medium consisted of the following ingredients: peptone water (2 g/liter), yeast extract (2 g/liter), NaCl (0.1 g/liter), K_2_HPO_4_ (0.04 g/liter), KH_2_PO_4_ (0.04 g/liter), MgSO_4_·7H_2_O (0.01 g/liter), CaCl_2_·6H_2_O (0.01 g/liter), NaHCO_3_ (2 g/liter), Tween 80 (2 ml/liter), hemin (0.05 g/liter), vitamin K (10 μg/liter), L−cysteine hydrochloride (0.5 g/liter), and bile salts (sodium glycocholate and sodium taurocholate) (0.5 g/liter). Each carbohydrate was added at the concentration of 1% (wt/wt) just prior to the addition of the fecal inoculum. Three vessels with no added carbohydrate source were included as negative controls. Temperature and pH were kept at levels that mimicked those of the distal region of the human large intestine. Thus, the temperature was held at 37°C by the use of a circulating water bath connected to each fermentation vessel; pH was maintained in the range of 6.6 to 7.0 via the use of pH controllers (Fermac 260; Electrolab, United Kingdom) and was automatically adjusted by adding 0.5 M NaOH or 0.5 M HCl to the vessels when required. The fermentation of each carbohydrate source was carried out in triplicate with the samples from each of three healthy human fecal donors. Subjects were free of any metabolic or gastrointestinal disorder, and they had not taken probiotic or prebiotic supplements or antibiotics for six months prior to the study. Fecal samples were collected and immediately placed in an anaerobic jar (AnaeroJar; Oxoid) (2.5 liters) equipped with a gas-generating kit (AnaeroGen; Oxoid). Samples were diluted at 10% (wt/wt) in phosphate-buffered saline (PBS) (0.1 M, pH 7.4) and homogenized in a stomacher (Stomacher 400; Seward, United Kingdom) for 2 min at 260 paddle beats/min.

A 2-ml volume of the fecal slurries (1% [vol/vol]) was used to inoculate the vessels containing the anaerobic fermentation medium and treatments. Batch culture fermentations were carried out for 24 h, and samples were collected at 0, 5, 10, and 24 h for analysis of bacterial populations and organic acids.

### Enumeration of bacterial populations by fluorescence *in situ* hybridization (FISH) combined with flow cytometry (FISH-FCM).

Differences in bacterial population numbers were assessed by FISH-FCM using 16S rRNA targeted oligonucleotide probes (Table S2). All probes were labeled at the 5′ end with fluorescent Cy3 dye (Sigma-Aldrich). For each of the time points, 375 μl of sample was mixed with three volumes of ice-cold 4% (wt/vol) paraformaldehyde (PFA) solution and fixed for 4 h at 4°C followed by centrifugation at 13,000 × *g* for 5 min. The cell pellet was washed twice in 1 ml sterile PBS and then resuspended in 300 μl of a solution consisting of sterile PBS-absolute ethanol (1:1 [vol/vol]). Samples were stored at −20°C until analysis. For the hybridization, 50 μl of each PFA-fixed batch culture sample was washed in PBS and treated with 1 mg/ml lysozyme–100 mM Tris-HCl–0.05 mM EDTA) (pH 8.0) at room temperature for 10 min to permeabilize the cells for use with the probes. Cells were washed in PBS and then resuspended in 1 ml hybridization buffer (5 M NaCl, 1 M Tris-HCl [pH 8], 30% formamide, double-distilled water [ddH_2_O], 10% SDS). An aliquot of 50 μl of sample was added to an Eppendorf tube with 4 μl of each probe (Table S2) and 4 μl of EUB338 I-II-III (linked to Alexa488). Hybridization was carried out at 35°C overnight. For the washing step, cells were pelleted by centrifugation, resuspended in 200 μl of washing buffer solution (5 M NaCl, 1 M Tris-HCl [pH 8], 0.5 M EDTA [pH 8], ddH_2_O, 10% SDS), and incubated for 20 min at 37°C. The pellet was then collected by centrifugation, suspended in 300 μl PBS, and stored at 4°C until flow cytometry (BD Accuri C6 Plus; Becton, Dickinson Biosciences, NJ, USA). Numbers of specific and total bacteria were determined, taking into account the dilution factor calculated from different volumes used in the steps of sample preparation, and the numbers of events per microliter were obtained from averaged numbers from NON EUB338 and EUB338 I-II-III probes (Table S2) analyzed by flow cytometry.

10.1128/mSphere.00554-18.7 TABLE S2 Oligonucleotide probes used in this study for FISH-FCM enumeration of bacterial populations. Download Table S2, DOCX file, 0.03 MB. Copyright © 2019 La Rosa et al. 2019 La Rosa et al. This content is distributed under the terms of the Creative Commons Attribution 4.0 International license.

### Organic acid analysis.

Acetic, propionic, and butyric acid concentrations were quantified using a gas chromatograph analyzer equipped with a flame ionization detector (GC-FID) following the method described by Richardson et al. (67) with ethyl butyric acid used as an internal standard. A GC analyzer (Agilent/HP 6890) equipped with an HP-5MS column (30 m × 0.25 mm) with a 0.25-μm-pore-size coating (cross-linked 5% phenyl methylpolysiloxane; Hewlett Packard, United Kingdom) was employed. Helium was used as the carrier gas at a flow rate of 1.7 ml/min (head pressure, 133 KPa). The oven initial temperature was set at 63°C followed by a temperature ramp of 15°C/min to 190°C and was then held constant for 3 min. A split ratio of 100:1 was used. Quantification of SCFA in the chromatograms was confirmed based on the retention times of the respective commercial SCFA standards (Sigma-Aldrich, United Kingdom) at concentrations ranging between 2 to 10 mM.

### Statistical analysis.

A paired Student's *t* test was used to evaluate significant differences among bacterial numbers and organic acid concentrations at the time of inoculation and the different sampling points. Data are presented as means ± standard deviations. Differences were considered statistically significant at *P* values of *<*0.05.
