# Supplementary material for: Wood-Derived Dietary Fibers Promote Beneficial Human Gut Microbiota
Source: mSphere. 2019 Jan 23;4(1):e00554-18. doi: 10.1128/mSphere.00554-18 (PMC6344601; doi:10.1128/mSphere.00554-18)
Supplement: TABLE S1 [file mSphere.00554-18-st001.pdf]

Table S1

Strain	Origin	Geographical location	Bioproject accession	β-Mannan PUL(s)	Xylan PUL(s)	Max OD_{600} on MM+Glc
Bacteroides						
B. finegoldii DSM 17565	Large intestine	Japan	PRJNA27823	None	None	1,44
B. caccae ATCC 43185	Large intestine	USA	PRJNA18163	None	None	0,83
B. cellulosilyticus CLT02T12C19	Large intestine	USA	PRJNA64803	HMPREF1062_00480-00518/ HMPREF1062_05286-05291	HMPREF1062_00480-00518/ HMPREF1062_03299-03311	1,42
B. cellulosilyticus DSM 14838	Large intestine	USA	PRJNA30027	BACCELL_02371-02385/ BACCELL_02270-02279/ BACCELL_03406-3426	BACCELL_03406-03426/ BACCELL_05685-05696	1,47
B. cellulosilyticus WH2	Large intestine	USA	PRJNA183545	BcellWH2_02020-02034/ BcellWH2_03032-03042/ BcellWH2_04292-04327	BcellWH2_04296-04327	1,46
B. cellulosilyticus WH206	Large intestine	NA	NA	NA	NA	1,55
B. clarus YIT 12056	Large intestine	Japan	PRJNA48509	HMPREF9445_02239-02254	None	1,25
B. dorei WH104	Large intestine	NA	NA	NA	NA	1,41
B. dorei DSM 17855	Large intestine	USA	PRJNA27831	BACDOR_00577-00585	BACDOR_00521-00543	1,44
B. eggerthii 1_2_48FAA	GIT	USA	PRJNA40009	HMPREF1016_03361-03378	HMPREF1016_02143-02174	1,46
B. fragilis NCTC 9343	Abscesses	UK	PRJNA46	BF0758-0774	None	1,48
B. fragilis VPI-499	NA	NA	NA	NA	NA	1,08
B. fragilis VPI-6779	NA	NA	NA	NA	NA	1,54
B. intestinalis DSM 17393	Large intestine	Japan	PRJNA20523	BACINT_00540-00556	BACINT_02803-02818/ BACINT_04193-04223	1,41
B. massiliensis DSM 17679	Blood culture	France	PRJNA169707	None	None	1,57
B. massiliensis A03	NA	NA	NA	NA	NA	0,18
B. nordii CLT02T12C05	Large intestine	USA	PRJNA64823	None	None	1,42
B. nordii WAL7936	NA	NA	NA	NA	NA	1,73
B. oleiciplenus YIT 12058	Large intestine	USA	PRJNA46377	HMPREF9447_01806-01817	HMPREF9447_01451-01476	1,35
B. ovatus 3_8_47FAA	GIT	USA	PRJNA40011	HMPREF1017_00342-00357/ HMPREF1017_02824-02840	HMPREF1017_02103-02113/ HMPREF1017_02791-02812	1,58
B. ovatus ATCC 8483	GIT	USA	PRJNA18191	BACOVA_02087-02097/ BACOVA_03386-03406	BACOVA_03418-03450/ BACOVA_04385-04394	1,48
B. ovatus VPI-3049	NA	USA	NA	NA	NA	1,80
B. ovatus WH604	NA	NA	NA	NA	NA	1,66
B. plebeius DSM 17135	Large intestine	Japan	PRJNA27829	BACPLE_03769-03780	BACPLE_01960-01983	1,81
B. thetaiotaomicron VPI-5482	Feces/Peritonitis	USA	PRJNA399	None	None	1,53
B. thetaiotaomicron 7330	GIT	USA	PRJEB8679	None	None	1,44
B. thetaiotaomicron WH3	GIT	USA	PRJNA227981	None	None	1,57
B. uniformis ATCC 8492	Large intestine	USA	PRJNA18195	BACUNI_00369-00395	None	0,90
B. uniformis WH504	NA	NA	NA	NA	NA	1,01
B. vulgatus ATCC 8482	Feces/Peritonitis	USA	PRJNA13378	None	BVU_0032-0044	1,47
B. vulgatus WH14	NA	NA	NA	NA	NA	1,44
B. xylanisolvens XB1A	GIT	USA	PRJNA39177	BXY_21910-02207/ BXY_28900-02960	BXY_29170-29440	1,51

Table S1. continued.

Strain	Origin	Geographical location	Bioproject accession	β-MOS utilization loci	XOS utilization loci	Max OD_{600} on SDM+Glc
Bifidobacterium						
B. adolescentis ATCC 15703	Intestine of Infant	NA	PRJNA16321	BAD_1030	BAD_0422-0430	1,47
B. animalis subsp. *lactis* Bl-04	Human Feces	NA	PRJNA32897	Balac_0013	balac_0511-0521	4,37
B. breve ATCC 15700	Intestine of Infant	NA	PRJDB770	No GH26	None	1,31
B. dentium ATCC 27534	Dental caries	NA	PRJNA237576	BBDE_1360	BBDE_0535-0548	1,83
B. longum subsp. *longum* ATCC 15707	Large intestine	NA	PRJNA224116	No GH26	None	0,43
Lactobacillus				**Short chain β-MOS and XOS utilization loci**		
L. acidophilus ATCC 4356	Human	NA	PRJNA222257	LBA0724-0727 + LBA0676		0,95
L. brevis ATCC 14869	Human Feces	NA	PRJNA222257	HMPREF0495_01584 (GH43) + HMPREF0495_00592 (GH51_1) + HMPREF0495_02112 (GH51_2)		0,9
L. gasseri ATCC 33323	Human	NA	PRJDB635	LGAS_1668-1670 + LGAS_1328		1,43
L. helveticus ATCC 15009	Cheese	NA	PRJNA34619	FC11_GL001642-44 + FC11_GL001191		1,73
L. plantarum WCFS1	Human saliva	Italy	PRJNA356	lp_3525-3529 + lp_0754		4,37
L. reuteri ATCC 23272	Large intestine	NA	PRJNA222257	No PTS gene/GH1 + FC53_GL000370		2,26
