# Supplementary figures and images for: Wood-Derived Dietary Fibers Promote Beneficial Human Gut Microbiota
Source: mSphere. 2019 Jan 23;4(1):e00554-18. doi: 10.1128/mSphere.00554-18 (PMC6344601; doi:10.1128/mSphere.00554-18)

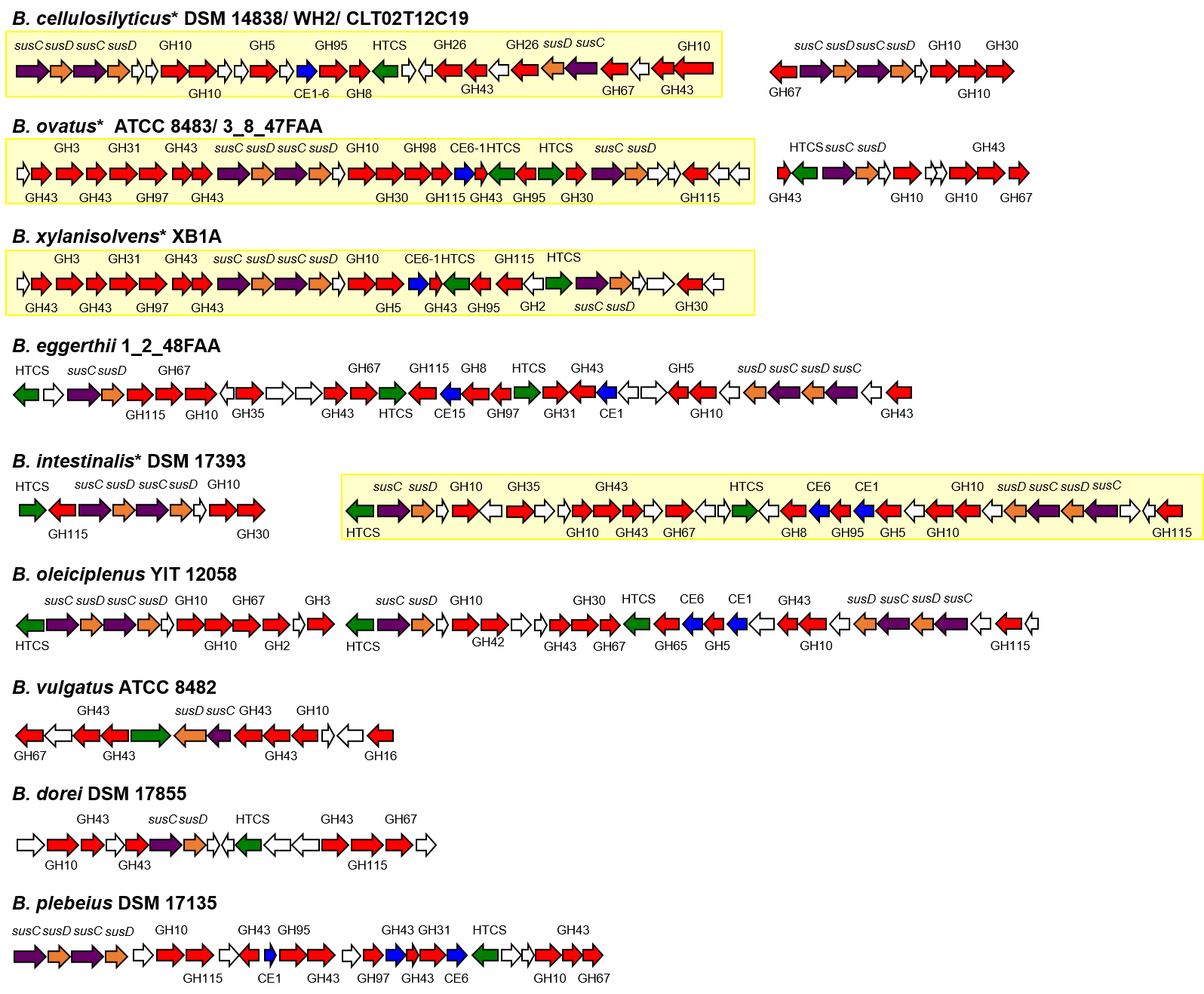

Supplement: FIG S1 [file mSphere.00554-18-sf001.tif]

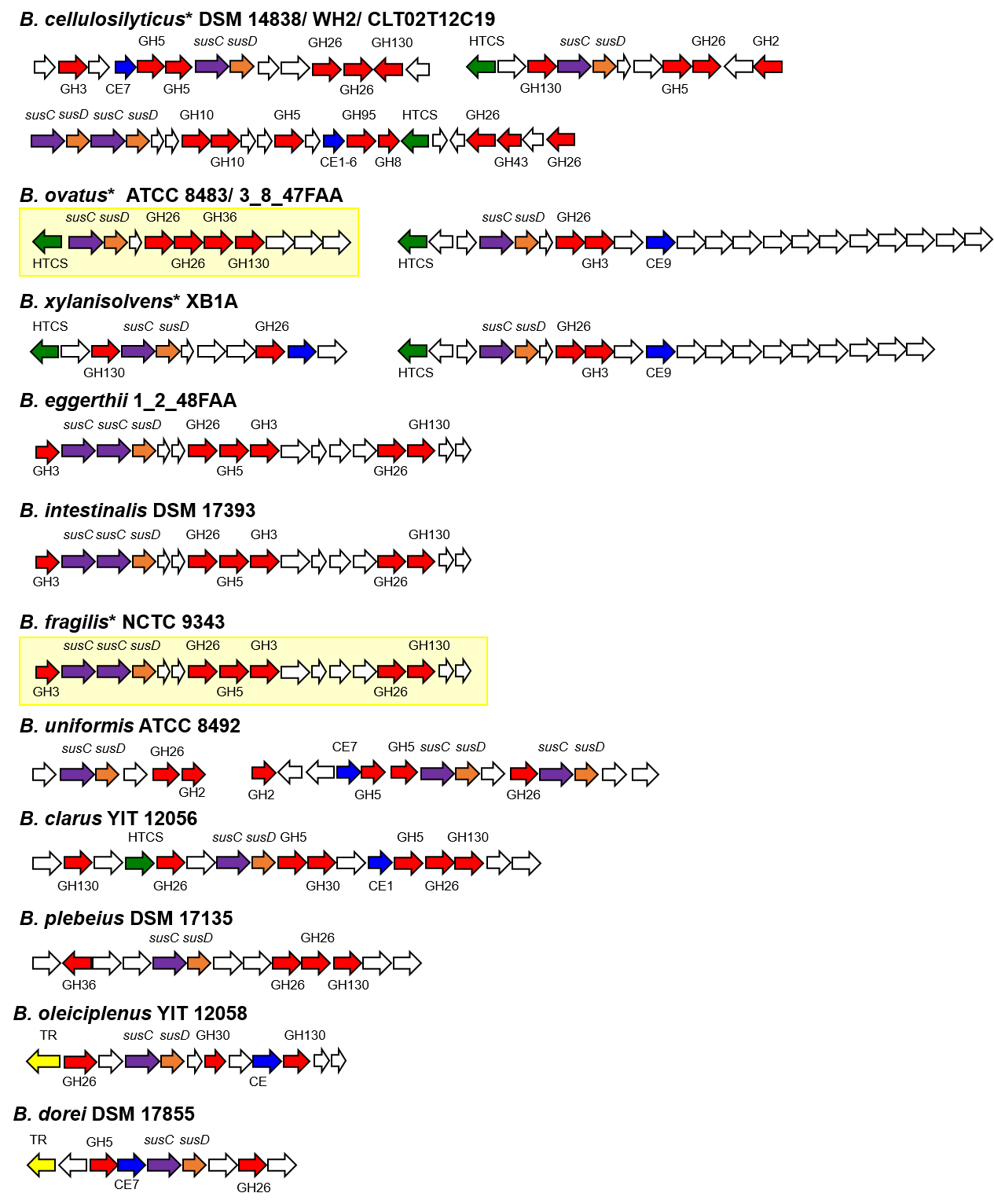

Supplement: FIG S2 [file mSphere.00554-18-sf002.tif]

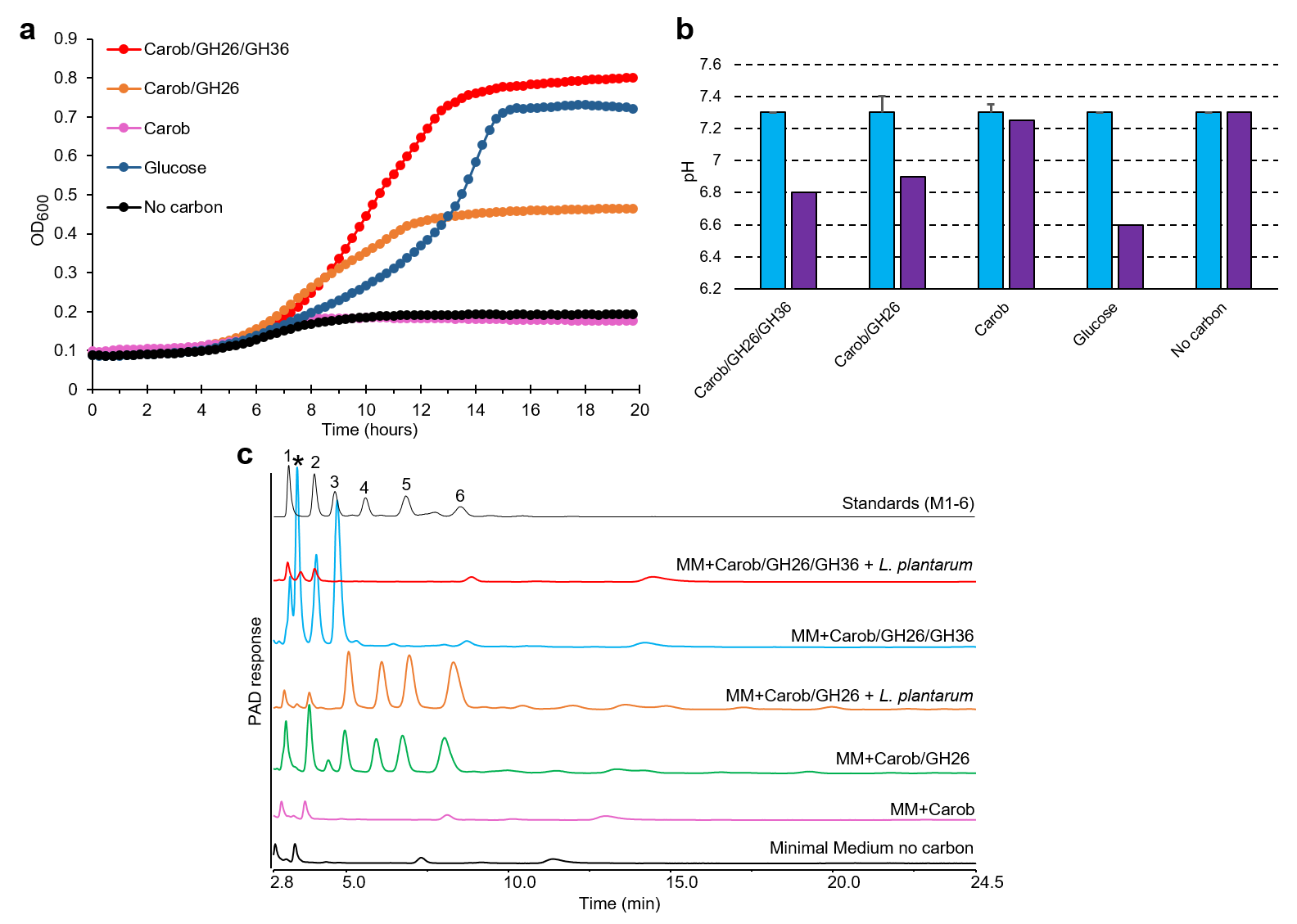

Supplement: FIG S4 [file mSphere.00554-18-sf004.tif]

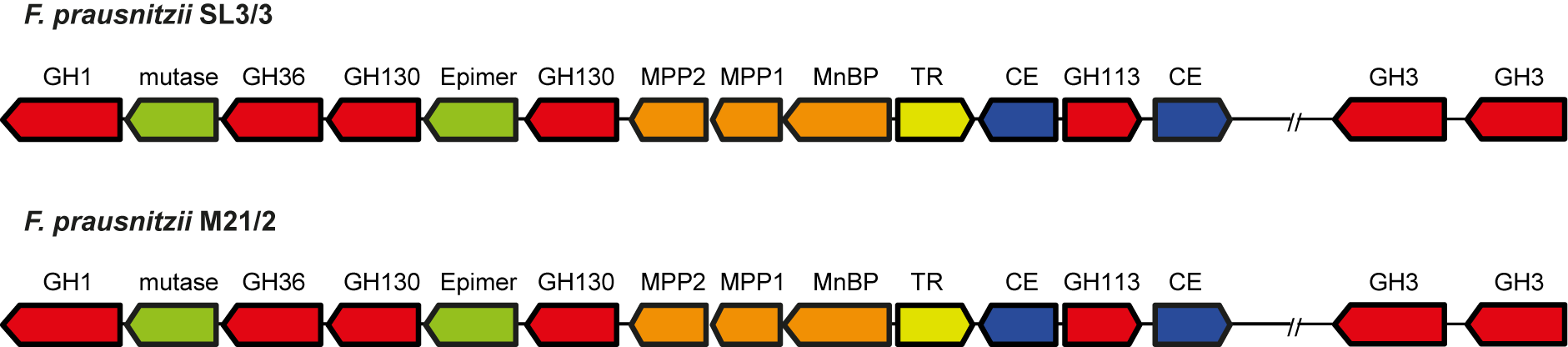

Supplement: FIG S5 [file mSphere.00554-18-sf005.tif]
