# Supplementary material for: Wood-Derived Dietary Fibers Promote Beneficial Human Gut Microbiota
Source: mSphere. 2019 Jan 23;4(1):e00554-18. doi: 10.1128/mSphere.00554-18 (PMC6344601; doi:10.1128/mSphere.00554-18)
Supplement: FIG S3 [file mSphere.00554-18-sf003.pdf]

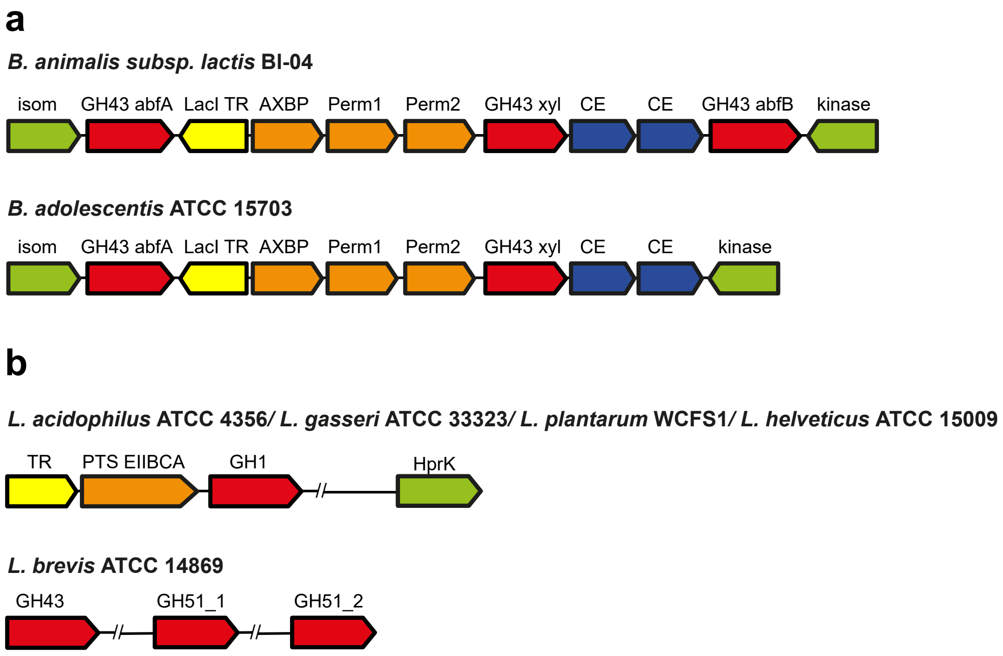
a
B. animalis subsp. lactis Bl-04
isom
GH43 abfA
LacI TR
AXBP
Perm1
Perm2
GH43 xyl
CE
CE
GH43 abfB
kinase
B. adolescentis ATCC 15703
isom
GH43 abfA
LacI TR
AXBP
Perm1
Perm2
GH43 xyl
CE
CE
kinase
b
L. acidophilus ATCC 4356/ L. gasseri ATCC 33323/ L. plantarum WCFS1/ L. helveticus ATCC 15009
TR
PTS EIIBCA
GH1
HprK
L. brevis ATCC 14869
GH43
GH51_1
GH51_2
