# Supplementary material for: Wood-Derived Dietary Fibers Promote Beneficial Human Gut Microbiota
Source: mSphere. 2019 Jan 23;4(1):e00554-18. doi: 10.1128/mSphere.00554-18 (PMC6344601; doi:10.1128/mSphere.00554-18)
Supplement: TABLE S2 [file mSphere.00554-18-st002.docx]

# **Table S2.** Oligonucleotide probes used in this study for FISH-FCM enumeration of bacterial populations.

| Probe name | Sequence | Target group | Reference |
| --- | --- | --- | --- |
| NON EUB | ACTCCTACGGGAGGCAGC |  | Wallner *et al.* (1) |
| EUB338 I | GCTGCCTCCCGTAGGAGT | Total bacteria | Daims *et al.* (2) |
| EUB338 II | GCAGCCACCCGTAGGTGT | Planctomycetales | Daims *et al*. (2) |
| EUB338 III | GCTGCCACCCGTAGGTGT | Verrucomicrobiales | Daims *et al*. (2) |
| BIF164 | CATCCGGCATTACCACCC | *Bifidobacterium* spp. | Langendijk *et al*. (3) |
| LAB158 | GGTATTAGCAYCTGTTTCCA | *Lactobacillus-Leuconostoc-Enterococcus* spp. | Harmsen *et al.* (4) |
| BAC303 | CCAATGTGGGGGACCTT | *Bacteroides*-*Prevotella* spp. | Manz *et al.* (5) |
| EREC482 | GCTTCTTAGTCARGTACCG | *Clostridium coccoides-Eubacterium rectale* group (*Clostridium* clusters XIVa and XIVb) | Manz *et al*. (5) |
| RREC584 | TCAGACTTGCCGYACCGC | *Roseburia* spp. | Walker *et al.* (6) |
| CHIS150 | TTATGCGGTATTAATCTYCCTTT | *Clostridium histolyticum* group  (Clostridium clusters I and II) | Franks *et al*. (7) |
| FPRAU655 | CGCCTACCTCTGCACTAC | *Faecalibacterium prausnitzii* cluster | Hold *et al.* (8) |
| ATO291 | GGTCGGTCTCTCAACCC | *Atopobium cluster* | Harmsen *et al*. (4) |
| PROP853 | ATTGCGTTAACTCCGGCAC | Clostridial cluster IX | Walker *et al.* (6) |
| DSV687 | TACGGATTTCACTCCT | *Desulfovibrionales* and  *Desulfuromonales* | Devereux *et al.* (9) |

1. Wallner G, Amann R, Beisker W. 1993. Optimizing fluorescent in situ hybridization with rRNA-targeted oligonucleotide probes for flow cytometric identification of microorganisms. Cytometry 14:136-43.

2. Daims H, Bruhl A, Amann R, Schleifer KH, Wagner M. 1999. The domain-specific probe EUB338 is insufficient for the detection of all Bacteria: development and evaluation of a more comprehensive probe set. Syst Appl Microbiol 22:434-44.

3. Langendijk PS, Schut F, Jansen GJ, Raangs GC, Kamphuis GR, Wilkinson MH, Welling GW. 1995. Quantitative fluorescence in situ hybridization of Bifidobacterium spp. with genus-specific 16S rRNA-targeted probes and its application in fecal samples. Appl Environ Microbiol 61:3069-75.

4. Harmsen H, Elfferich P, Schut F, Welling GW. 1999. A 16S rRNA-targeted probe for detection of lactobacilli and enterococci in faecal samples by fluorescent in situ hybridization. Microb Ecol Health D 11:3-12.

5. Manz W, Amann R, Ludwig W, Vancanneyt M, Schleifer KH. 1996. Application of a suite of 16S rRNA-specific oligonucleotide probes designed to investigate bacteria of the phylum cytophaga-flavobacter-bacteroides in the natural environment. Microbiology 142 ( Pt 5):1097-106.

6. Walker AW, Duncan SH, McWilliam Leitch EC, Child MW, Flint HJ. 2005. pH and peptide supply can radically alter bacterial populations and short-chain fatty acid ratios within microbial communities from the human colon. Appl Environ Microbiol 71:3692- 700.

7. Franks AH, Harmsen HJ, Raangs GC, Jansen GJ, Schut F, Welling GW. 1998. Variations of bacterial populations in human feces measured by fluorescent in situ hybridization with group-specific 16S rRNA-targeted oligonucleotide probes. Appl Environ Microbiol 64:3336-45.

8. Hold GL, Schwiertz A, Aminov RI, Blaut M, Flint HJ. 2003. Oligonucleotide probes that detect quantitatively significant groups of butyrate-producing bacteria in human feces. Appl Environ Microbiol 69:4320-4.

9. Devereux R, Kane, MD, Winfrey, J, Stahl, DA. 1992. Genus- and group-specific hybridisation probes for determinative and environmental studies of sulphate-reducing bacteria. Syst Appl Microbiol 15:601-609.
